# Trauma in Animal Protection and Welfare Work: The Potential of Trauma-Informed Practice

**DOI:** 10.3390/ani12070852

**Published:** 2022-03-29

**Authors:** Rochelle Stevenson, Celeste Morales

**Affiliations:** 1 Department of Sociology and Anthropology, Thompson Rivers University, Kamloops, BC V2C 0C8, Canada; 2 Vancouver Humane Society, Vancouver, BC V6P 5A2, Canada; celeste@vancouverhumanesociety.bc.ca

**Keywords:** trauma-informed practice, animal protection, animal welfare, compassion fatigue, burnout, qualitative method, interviews

## Abstract

**Simple Summary:**

Professions that involve caring for others can present emotionally challenging and traumatic situations that may have negative effects on a worker’s mental health. This is also true for workers in the animal protection sector, particularly for those who participate in animal surrenders and removals, and witness both human and animal suffering. Changes are needed to mitigate and prevent these negative effects. To explore the challenges experienced by animal protection and welfare workers and better understand the changes that need to be made, we interviewed 11 individuals who work in the animal protection and welfare sector and have experience with the surrender and/or removal of animals. We found that the participants experienced many challenges that affected their mental health. We recommend that trauma-informed practices be implemented in the animal protection and welfare sector in order to manage and prevent job-related stress and trauma. We suggest that trauma-informed practices will help to develop both individual and organizational resilience, and result in a more compassionate experience for both workers and animal guardians.

**Abstract:**

Those who work in the animal protection and welfare (APW) sector are consistently exposed to human and animal suffering, particularly those who witness animal surrenders and seizures. Continued exposure to suffering can result in stress, anxiety, burnout, and compassion fatigue, which are detrimental to individual and organizational well-being. The aim of this study was to understand the challenges experienced by Canadian APW workers, and to explore how trauma-informed approaches can be implemented to help mitigate these challenges. To achieve this, we utilized purposive sampling to seek workers in the APW sector who had experience with animal surrender and/or seizure. Telephone interviews were conducted with 11 participants. Participants reported experiencing many challenges that negatively impacted their mental health; this article summarizes them by focusing on two key themes drawn from the narratives of the participants: feeling unprepared and forced strength. Trauma-informed practices are explored as a means to prevent compassion fatigue and burnout, and to increase the resilience of individuals and organizations. We suggest trauma-informed practices help APW workers manage job-related stressors while also providing a more compassionate experience for animal guardians. Further, we propose that trauma-informed practices are a crucial component in facilitating respectful relationships with the communities that APW organizations serve.

## 1. Introduction

The animal services sector is a diverse space, encompassing animal shelters, animal protection and enforcement, municipal bylaw enforcement, on-reserve animal by-law officials or portfolio holders, SPCAs, humane societies, animal sanctuaries, rescues, and veterinary services. A challenging feature of the Canadian animal protection and welfare sector is that many organizations are nonprofits, meaning that they rely primarily on charitable donations to fund their operations [1]. As with other non-profit sectors, animal protection and welfare organizations rely on a mix of paid staff and volunteers to fulfill their mandates. There is overlap in mandates and activities within the animal protection and welfare sector. Animal protection typically includes peace officers and investigators whose mandates involve the investigation of complaints and enforcement of animal-related legislation. This protection and investigation work rests largely on the shoulders of non-profit animal welfare organizations that also engage in sheltering and rescuing activities [1].

While recognizing the diversity of roles and organizations within the animal protection and welfare sector, our research focuses on paid workers in animal shelters, animal protection and enforcement, and animal welfare and rescue organizations. We refer to animal protection and welfare (APW) together in this paper to acknowledge the voices of our participants from these overlapping spaces. 

We recognize there is a larger literature on the performance of emotions in workplaces, what is known as emotional labour. Professions that involve caring for others, whether it be animals or humans, inherently encounter emotionally challenging situations. Those who work in helping roles are seen as being at a greater risk of developing “stress-related conditions such as depression, anxiety, and compassion fatigue” [2] (p. 1). Compassion fatigue is the emotional exhaustion and reduced compassion that can result from chronically using empathy when helping those who are suffering [3,4].

Compassion fatigue has been documented in helping professions such as nursing, counselling, social work, family services, and policing. In the nursing sector, for example, workers are at risk of compassion fatigue due to their consistent proximity to tragedy, exposure to suffering and stress, lack of support, and lack of self-care, amongst other challenges [5]. Correctional workers, firefighters, and paramedics, along with support personnel such as dispatchers, report high exposure to traumatic experiences as part of their daily job, and such exposures have negative impacts on mental health [6,7]. Police officers also witness trauma and victimization as part of their day-to-day work, ranging from automobile accidents to victims of interpersonal violence [8,9,10]. Research has illustrated that the stress of the job added to the “costs of caring” for victims increases compassion fatigue among police officers [8,11,12]. Those in the social work sector have similar risk factors for developing compassion fatigue, including providing support to those who have experienced abuse and violence, experiencing emotionally challenging situations, and the general stressful nature of the profession coupled with a deep connection to and passion for the work [13].

Similarly, workers in the APW sector are often exposed to animal suffering when caring for sick or injured animals [14,15,16]. Workers may also provide services to human guardians who are emotionally distressed, or grieve with guardians who experience trauma, often on-going, in their lives. Removal of animals (seizure) and surrender are two aspects of APW work where workers observe both animal and human suffering. Animal seizures are typically the result of neglect or violence against the animal, and the animal is legally removed from the owner or guardian. Animal surrenders occur when the guardian can no longer care for the animal and surrenders legal ownership of the animal to an APW organization. Surrenders are always voluntary—some are freely chosen by the guardian, while others are a more difficult decision driven by changing life circumstances or via coaching by APW workers.

Both seizure and surrender can take an emotional toll on the worker and result in negative effects on their well-being. In the personal context, workers in the APW sector have reported “high levels of exhaustion, relationship conflict, poor well-being, sadness, sleep difficulties, and feelings of guilt and anger resulting from their work” [2] (p. 1), [14]. In the organizational context, experiencing compassion fatigue can result in “increased absenteeism, reduced well-being, poor work satisfaction, and poor staff turnover” [2] (p. 1). 

In addition to compassion fatigue, the nature of APW work means that workers are also at risk of experiencing burnout, which results from chronic work-related stress. Work related-stresses can be differentiated into organizational stress (e.g., limited resources, heavy caseloads, long hours, and shift work) and operational stress (stress and trauma from conducting the work) [9,12]. The main symptoms of burnout are emotional and physical exhaustion, cynicism or feeling detached from one’s job, a reduced sense of accomplishment, and feeling inadequate [17,18]. Burnout has been explored in the context of helping and caring professions, including law enforcement [11,12,19,20]. Organizational difficulties that APW workers face in their line of work increase stress and contribute to burnout, such as difficult working conditions and coworker dynamics, a lack of training and support, poor management, and challenges related to external conditions such as a lack of funding, local legislation, and public apathy [15,16,21]. Animal protection officers have reported how others underestimate, undervalue, and demean the work of APW, viewing APW workers as either glorified ‘dogcatchers’ or ‘unreasonable extremists’ [15]. Both of these conceptualizations were in contrast with how APW workers viewed themselves, and were discouraging to those already performing a difficult job [15]. Rault and colleagues document several emotional challenges in APW work, such as witnessing cases of extreme cruelty and threats of violence and actual physical harm to APW workers [16]. The emotional situations and suffering that APW workers are involved in and exposed to, paired with workplace stressors such as large caseloads, emergency calls, aggressive animals, challenging interactions with people requiring animal services, and environmental hazards [15,16,22], place APW workers at risk for experiencing both compassion fatigue and burnout [23]. 

Though compassion fatigue and burnout have been explored in the context of other caring professions [19,24,25], only recently has research begun to explore the prevalence of compassion fatigue among animal service workers [2,22,26,27,28,29]. Furthering this line of inquiry is essential as findings have shown that APW workers “face consequences related to their jobs, encompassing a variety of mental and physical health problems” such as high blood pressure, depression, and substance use [28] (p. 96).

Experiencing compassion fatigue, burnout, and their related effects is not only difficult for the individual, but can also have impacts beyond the individual. Compassion fatigue and burnout may negatively impact how a worker interacts with people accessing services, which can “interfere with a worker’s ability to provide an emotionally safe environment, increasing the risk of the person accessing services being further traumatized and compromising animal safety” [18] (p. 22). As such, this topic is worthy of further exploration, in order to help create more emotionally safe work environments for animal service workers and, in turn, better outcomes for animals and their guardians.

The concept of One Welfare acknowledges these many interconnections between animal and human well-being and the environment [30]. The well-being of one affects the other, and is affected by the other. It is a “collaborative approach for integrating animal welfare, human well-being and the environment” [31] (p. 12). Relevant to the current study, the One Welfare framework also acknowledges the links between animal and human abuse and neglect, that poor well-being in one sphere contributes to poor well-being in other spheres. Seizures and surrenders are directly related to the poor well-being of the animal as a result of abuse or neglect, but often include the poor well-being of the guardian [32,33]. The One Welfare framework includes the APW workers as well, in recognizing the secondary and compounding trauma of witnessing abuse and neglect.

Providing trauma-informed services is a method within the One Welfare framework that increases well-being for all. At its core, a trauma-informed approach acknowledges that trauma is a common experience; embracing this approach is a way to avoid causing more harm and more trauma to others, and to prioritize well-being for everyone involved. Wilson, Fauci, and Goodman clarify that trauma-informed practice (TIP) is not focused on treatment for the individual trauma, as this is often beyond the scope of human (and animal) service organizations [34]. TIP is instead centred on delivering services to clients in a way that is appropriate and sensitive to the unique needs of trauma survivors. Increasingly, the benefits of adopting a trauma-informed approach for service providers are being recognized [24,35], particularly in the fields of child welfare and protective services [20] and domestic violence services [34], as these are two fields in which secondary trauma for service providers is common. Research has begun to show that trauma-informed service settings achieve better outcomes, and show positive impacts on staff [24]. Hales and colleagues explored the impact of TIP on staff at a non-profit agency providing supports for mental health and substance use issues [36]. The study assessed agency staff before and after the implementation of TIP, finding that TIP increased staff satisfaction in almost all areas, including direction of the organization and agency values, relationships with colleagues and management, and their connection to the organization [36]. Key to these positive results was the active role that the organization took in implementing TIP.

TIP includes two elements: the individual, and the organization [34,37]. Trauma-informed systems and organizations “provide for everyone within that system or organization by having a basic understanding of the psychological, neurological, biological, social and spiritual impact that trauma and violence can have on individuals seeking support”, and further, “recognize that the core of any service is genuine, authentic and compassionate relationships” [38] (p. 16). A trauma-informed service provider, system, or organization also realizes the widespread impact of trauma and understands potential ways for healing, recognizes the signs and symptoms of trauma in staff, persons accessing services, and others involved in the system, and responds by incorporating knowledge about trauma into their policies, procedures, practices, and settings [38]. This underscores the comprehensive nature of TIP, and the depth to which both organizations and individuals must embrace trauma-informed principles.

There are six key principles of TIP:

1. “safety;
2. trustworthiness and transparency;
3. peer support;
4. collaboration and mutuality;
5. empowerment, voice and choice; and
6. cultural, historical, and gender issues” [37] (p. 10).

We address the fifth and sixth principles of empowerment, voice and choice, and cultural, historical, and gender issues comprehensively in a separate publication (see [18]). For this article, we will focus on the first four key principles.

Understanding that APW workers experience trauma in their work, it is important to consider how trauma-informed practices can be beneficial for workers involved in instances of animal seizure, surrender, and abuse. In their professional roles, APW workers provide support to people who have experienced severe trauma in their lives [39], and this can trigger feelings of helplessness, sadness, and anger. APW workers may also be confronted with threats or violence in their work or have their own histories of trauma, as has been noted in other human service sectors [19,40], and/or be impacted by intergenerational trauma [39].

An important aspect of trauma-informed practices is ensuring worker wellness and safety, both physical and emotional. Physical safety is widely governed by federal and provincial regulations. Canada’s *Occupational Health and Safety Regulations* focuses on physical hazards, such as exposure to hazardous materials and building safety. Provincial workplace regulations, such as in Ontario and British Columbia, have sections that address violence in the workplace with the onus on the employers to have policies in place to eliminate the risk of violence. However, as with the federal regulations, physical safety is the focus in the provincial regulations without mention of emotional safety. Trauma-informed approaches attend to both physical and emotional safety throughout an organization, with an awareness of the stress and trauma that workers may experience. TIP incorporates strong policies supporting comprehensive well-being, utilizes staff education and coaching, and models activities that support workers’ self-care [24,39]; in essence, it builds resilience to dealing with trauma. It is important to note the difference between resilience and coping, as this difference is integral to a true trauma-informed approach. Resilience is conceptualized as the positive solutions-based response to negative circumstances using problem-solving skills, supportive resources, and healthy coping practices [41]. Coping, on the other hand, is a component of resilience, but is limited to managing the emotions and stresses of the circumstance and may include unhealthy strategies [41]. An organization that embraces TIP prioritizes the more fulsome practice of building resilience among employees with policy and practice, rather than merely providing options for coping with job-related stresses.

Under the One Welfare framework, the use of trauma-informed practice benefits all involved in the animal welfare and protection sector, including front-line staff, animal guardians, and animals themselves. This article focuses on the humans working in APW by sharing their lived experiences and challenges, and highlights the necessity of implementing trauma-informed practice in APW work by demonstrating how trauma-informed approaches can mitigate and prevent these challenges.

## 2. Materials and Methods

This qualitative study employed an exploratory approach in order to understand the challenges APW workers face when engaging in surrender and seizure processes, and explore how a trauma-informed approach can be implemented in the APW sector to help mitigate these challenges. This study is a component of a larger project conducted by the Vancouver Humane Society [18], which comprised 28 interviews with individuals with lived experience of surrender or seizure of their animal companions, staff in non-animal-related organizations who incorporated trauma-informed practices, and workers in the APW sector. The study received ethical clearance from Thompson Rivers University (File #102608). This article focuses on the experiences of a subset of 11 participants: those who identified as workers in animal protection and welfare who have experience with the surrender and/or seizure of animals.

Purposive sampling was used as a technique to recruit interview participants. The interview participants for this study were found through Facebook ads, related social media posts, word-of-mouth, and existing personal and professional networks. All promotional communications invited potential participants to contact the research team if they were interested in participating. Within these communications directed at the animal services sector, we asked that potential participants meet the inclusion criteria of experience working in the APW field in Canada, with a minimum of 6 months of experience providing services related to the surrender or seizure of animals, either directly with seizure or surrender situations or with organizational policy related to seizure and surrender situations. The first ten participants to respond to the promotional communications who met the inclusion criterion were selected to participate in the study. One additional participant was included at a later date.

Participants came from an array of animal protection and welfare organizations from across Canada, and included animal investigation officers, front desk and support staff at animal shelters, non-profit rescue agency staff, and animal welfare agency leadership. All participants were paid staff, either currently employed in the APW sector or retired. At the outset of the interviews, the participants were asked about the race/ethnicity and gender with which they identified as a way to gain a better understanding of our participant sample. We share this demographic information as a means to provide transparency and context to the participants and their lived experiences. No further demographic information is shared in order to protect the participants’ confidentiality and identity, and participants are referred to with codes (e.g., P1). Throughout this paper, we use the language that the participants used to describe themselves. The participant group primarily identified as White (*n* = 10) with one person identifying as Mixed. Regarding gender identities, the APW group were mainly female identifying (*n* = 9) with one male and one non-binary participant.

All of the interviews were conducted over the phone, with the interviewer calling the participant at a scheduled time. All participants provided free and informed consent before the interview began, and were told that they could withdraw from the interview at any time during the interview, and up to 15 days after the interview. No participants withdrew from the study. As part of the informed consent process, participants were promised confidentiality, given that some of the questions could have resulted in critiques of the APW sector, or their workplace.

Each participant received a gift card valued at $50 CAD for their participation. Participants were given the option to review the transcript of their interview to decide whether they would like to remove or add any information. No participants changed or revised the transcripts. Participants were also provided with information about the research project before the interview began, were given the opportunity to ask questions or address concerns, and were told at the outset of the interview that they were welcome to skip questions and take breaks throughout the interview given the nature of the topics of discussion. At the end of the interview, participants were given the option to receive a list of mental health resources, which included culturally appropriate resources, in the event that the interview brought up any difficult emotions and feelings.

The interview questions were of a semi-structured and open-ended nature, and interviews lasted between one and three hours, with an average interview length of 86 min. The semi-structured nature of the interviews allowed for consistency in terms of the themes covered within the interviews, but also allowed flexibility and freedom for participants to discuss issues of concern or aspects of their work that they felt important. For an overview of the questions that were used to guide the interviews, please see Appendix A (also see [18]).

The interviews were fully transcribed using the Wreally digital transcription website, and were confirmed by the two principal investigators and a research assistant. A thematic analysis approach was employed to analyze the interview transcripts, drawing on the process proposed by Braun and Clarke [42,43]. The transcripts were analyzed for themes derived from the research literature (e.g., symptoms of compassion fatigue and burnout, and trauma-informed practices), themes suggested by the research questions (e.g., work stresses and ideas for change from participants), and those that arose organically from multiple and iterative readings (e.g., feeling unprepared). While the two themes discussed below encompass varied experiences of the participants, they do reflect shared meanings of participants in the challenges of their jobs and the possibilities of TIP.

## 3. Results

While there were many ideas that arose from the interviews with participants, this article focuses on two key themes drawn specifically from the narratives of the APW participants: Feeling Unprepared and Forced Strength. Both of these themes offer evidence for the necessity of implementing TIP within the APW sector.

### 3.1. Feeling Unprepared

Within the theme of Feeling Unprepared, the voices of the participants included two connected issues, namely inadequate preparation for trauma seen as part of day-to-day work and a lack of training resulting in inadequate skills to handle conflict.

#### 3.1.1. Inadequate Preparation for Realities of Work

The theme of feeling inadequately prepared for the day-to-day realities of the job arose in a few participant interviews; it is an important issue when considering TIP. Feelings of unpreparedness for the emotional impacts of the work, the degree of animal and human trauma witnessed during conversations around surrender, or situations that required seizure were raised by participants. Even though some of the participants had experience in service professions, and all had the desire to help animals, there was, as P7 framed it, a feeling of shock when witnessing the “in your face” suffering of the animals, and their guardians. This shock and trauma was not exclusive to organization staff, but included volunteers as well. P7 shared that while there was an orientation book for organization volunteers, it did not prepare them for the circumstances of animals and guardians that they encountered. When talking about seizures of large numbers of animals, the frustration and helplessness of P1 were apparent in her words: “I’m not a doctor. I’m nobody, but I obviously see mental illness” evidenced by the deplorable conditions from which the animals were being removed. As with P7, P1 received little training or acknowledgement of the impact on staff of attending these calls.

TIP can offer important tools here for APW workers, primarily the acknowledgement of the secondary trauma that can affect staff who witness the trauma of animals and their guardians, or unwillingly contribute to such trauma via seizures. First, having open conversations about these possible situations in advance can mitigate the negative effects by clearly acknowledging that secondary trauma is possible, which serves to mitigate the potential stigma of poor mental health. Second, purposive training using scenarios can help staff to work though potential emotions that may arise in a safe space. This style of training not only introduces APW staff to the realities of their work, but also undertakes this in a secure environment and in a way that validates the emotional and psychological responses of the staff. Within this scenario-based training, tools to manage emotions in the moment should be introduced, and reiteration of the support services available after tough calls should be emphasized.

Part of feeling unprepared for the realities of APW work was a recognition of the structural and resource-related factors that impacted the APW workers’ ability to conduct their jobs. There was a degree of frustration that shone through, rooted in the overwhelming workload, at the lack of resources within the agency and within the community, and the perceived inability to improve resources for humans and animals.

P7 commented that “you start off with big plans, big ideas, but then on the ground, you’re just jumping from emergency to emergency, and it is hard to get past the crisis level […] I spend a lot of my time trying to get through the day-to-day things”. The ability to feel prepared is hindered by the workload, and the need to deal with the seemingly never-ending crises that arise. Most participants shared that they worked in a constant state of reaction, and that proactively trying to address the causal and structural factors that led to surrender or seizure was challenging, and at times frustrating. P4 highlighted how the stress of this can spill over into interactions with other staff members, sometimes resulting in taking frustrations out on colleagues. When there is little preparedness for the level of need on the part of guardians, stress due to demands on time and resources, and a lack of training for stressful situations, this can create an unhealthy and unsafe work environment for staff, and can compound the effects of compassion fatigue and burnout.

The lack of access to resources experienced by animal guardians was an issue that came up in relation to the realities of the job. For example, P5 shared her early experiences in a remote community: “You have dogs that necessarily don’t have food, water, and shelter on a consistent basis, but then if you really look at the people, a lot of times the people don’t have those things either”. For P5, this built up her compassion, but also was a challenging reality to navigate when trying to adhere to animal-focused organization mandates and policies. A few participants specifically referenced the lack of geographically accessible veterinary care as a resource issue that was particularly salient in rural and remote areas. P6 and P7 both referenced the challenges of not having a regular veterinarian in the area, and the substantial travel time required to seek veterinary services. Even though in P7′s case the (more) local veterinarian was offering a mobile practice, which served some of the need, serious animal health or medical concerns that arose required travel, sometimes to other provinces.

While TIP will not solve the structural inequities and corresponding challenges in a community, it does offer approaches that can reduce the negative impact of APW work, such as collaboration between human and animal service organizations. One of the ways in which participants alluded to feeling more prepared was through collaboration with other community organizations, specifically human service organizations, to help address the needs of the guardians while they focused on the needs of the animals. This practice embodies a One Welfare approach, recognizing that helping guardians also helps the animals the APW organization serves. Identifying community need through collaborative and respectful conversations with the community, and then building relationships between complementary organizations to address the needs, is an important facet of TIP that benefits the guardians, animals, and APW staff. P1 succinctly gave voice to the feelings of helplessness that the situation would simply repeat itself, which was common to participants: “We’ve been here before”. Establishing formal or informal agreements between human and animal service organizations can provide information and pathways to both reactively and proactively support humans and animals. These trauma-informed actions can help to mitigate the feelings of helplessness and frustration on the part of the APW workers, which are key emotions that can lead to compassion fatigue and burnout.

#### 3.1.2. Inadequate Skills to Handle Conflict

One of the tough situations that several participants raised was conflict with human guardians. Increased training for managing conflict arose frequently as a theme. Insults, verbal abuse, threats, and even physical assaults were reported by participants. Most participants felt that they did not have the skills to handle such conflict, or if they did have some skills, these were not adequately developed in training provided by the organization. For example, P4 cited the lack of training of front-line staff, leaving them unprepared for “the kind of situations that you would encounter”, explaining that people are often in a “pretty rough situation” when coming to the organization for help, which sometimes results in stress-related conflict. P4 shared experiences of being sworn at, physically threatened, grabbed, and insulted, noting that their size and presumed male gender likely resulted in different interactions than their smaller-statured female or femme co-workers, who were also being intimidated and threatened. P4 clearly stated “we’re not really getting a lot of training to handle a lot of these high-stress situations”. P4′s sentiments were echoed by other participants, both in terms of the experience of verbal abuse as well as the recognition of the sensitive mental and emotional state of guardians who were in the process of the surrender or seizure of their animal, which is an incredibly emotionally challenging situation.

Most participants acknowledged that animal guardians who were surrendering their animals, or who were in the process of having their animals seized, were frequently not in a good space themselves, often dealing with mental health issues, challenges related to poverty, a lack of resources such as accessible veterinary care, and/or the continued impacts of intergenerational trauma. There was also the acknowledgement that there was a degree of mistrust and a fear of social service organizations connected to the continued impacts of systemic oppression. While some participants felt that they had developed skills in previous positions, all participants recommended increased training on how to better manage situations where conflict can arise. P9 suggested training in recognizing mental health crises, as this was a circumstance that was frequent in their experience: “the client is obviously very upset, and potentially dealing with their own mental health issues, and they bring up suicide. That’s something that’s quite serious, and obviously not only for the health of the animal, the safety of the animal in that circumstance, but also the health and safety of the person”. P9 further stated that there were times where it was “not safe or healthy” for them to deal with the upset or in-crisis clients on their own. Feelings of a lack of safety were shared by a few other participants, notably concerns around personal safety and situations of conflict, threats, and verbal abuse.

Professional development opportunities, such as communication skills or de-escalation training, as possible ways to build competencies in dealing with guardians in crisis was a frequent suggestion. This further evidenced the feeling of inadequate preparation to deal with the regularly occurring conflict with animal guardians. The suggestion of increased training is in line with TIP, in that on-going education and skill building will help to mitigate trauma for the human guardian, animal companion, and staff member by fostering trustworthiness through transparent and honest communication. In addition, TIP promotes communication with people accessing services in a way that is compassionate, non-judgmental, respectful, non-triggering, and centers a person’s voice and choice in the situation. This style of communication is beneficial for APW workers as it can help to build trust and rapport with people accessing services, resulting in a more positive service interaction for all involved. For example, P3 suggested that something as simple as improving communication skills increases staff motivation (which is a way to counter burnout), and allows staff to gain confidence to handle situations “with people that are not managing their animals” in a compassionate way, thus embracing a One Welfare approach.

On the other hand, P9 felt that the de-escalation training that had been provided was helpful, but not comprehensive enough to deal with the myriad situations that they encountered on a regular basis. Training grounded in TIP principles, such as active listening, meeting a person where they are at, and a One Welfare approach that connects the well-being of human and animals, can be a way to manage the negative feelings that can result from dealing with crisis after crisis. When APW workers feel that they are not helping human or animal, and feel that they are not making a difference in the lives of the beings they desire to help, this is a pathway to burnout. Modelling scenarios using practical examples and solution-focused strategies can help APW staff to feel empowered in their dealings with animal guardians. This also helps to build resilience among staff in strengthening problem-solving skills, which further supports the well-being of APW workers, human guardians, and animal clients.

### 3.2. Forced Strength

The theme of Forced Strength was voiced by participants in the perception of feeling overworked and unable to take needed time off (e.g., cannot say no to work), having to mask feelings of compassion fatigue, burnout, and mental health challenges, and having to balance professionalism and empathy (e.g., cannot show weakness on the job).

#### 3.2.1. Needed Time and Resources

Feeling overworked was a consistent topic in the interviews, often raised at multiple points, illustrating the impact on the overall well-being of the APW workers. While the issue of overwork, and its effect on preparedness, was addressed under the theme of Feeling Unprepared, it also appeared in a unique way within the theme of Forced Strength, as the perceived inability or unwillingness to take the needed time and resources to attend to self-care. Whether the implied strength was modelled by organization leadership, or self-imposed due to not wanting to burden co-workers, participants were clear that more attention and acknowledgement of mental health was required.

The trauma of difficult seizures, witnessing human and animal suffering, and conflict with guardians was compounded by the implied expectation that AP staff just ‘deal with it’ and not let it affect their work. This perceived strength was modelled by leadership in some cases, though some participants in leadership positions did recognize the impact of their actions. Despite supports being available in their organization, P2 acknowledged “we always say ‘do as I say not as I do’ which is bad…we are not walking the walk very well”. P5, a leader in her organization, shared that there were no formal supports in place, nor had any processes been initiated, just that the volunteers may get together and talk about the day. While keeping the lines of communication open for staff, P5 did not herself feel the need to debrief or destress as she could “handle it better than most”. The impression left is that the leaders or people in positions of authority are better able to handle the stress and secondary trauma than others; however, this models a behaviour that others may (and do) feel forced to emulate in their work, with negative consequences.

Some organizations, through a lack of adequate mental health supports, fostered an atmosphere of forced strength. P9 shared that their workplace did not really facilitate space for dealing with difficult circumstances, conveying that they did not feel able to “say ‘well that was really upsetting,’ or you know, ‘I really need to decompress’” or take the needed time to “go home and have a cry or have five minutes to go for a walk or something”. Later in the interview, P9 also shared that they did not feel as though they “could just take a mental health day” if they needed one. In this feeling of not being able to engage in self-care and rest, there is a forced strength that can contribute directly to burnout.

Other organizations offered mental health resources and supports such as mental health days, but there was a sense of guilt and shame in taking advantage of the resources, often related to workload. For example, P6 mentioned that if she was feeling burnt out, there was the option to take a few extra days off, but that she would not exercise this option as it would mean more work being placed on her co-worker’s shoulders. She also related that even with another person to help with the workload, she was “probably still not going to [take mental health days]”. There was a sense that if she did take mental health days, this meant that she could not handle the job. P10 also referenced workload as contributing to their compassion fatigue and burnout, stating “your salary’s based on 35 hours a week, but they [leadership] know damn well you’re doing 50 [hours]”. The implicit expectation, or at the very least lack of overt recognition, of overwork was a part of the perception that the worker should be strong enough to handle the emotional and physical demands of the job: “We were told there should be a work-life balance, but it wasn’t genuine […] you couldn’t show a bad day” (P10). The scarcity of labour resources forces a strength onto APW workers, as taking needed time may mean that animals do not receive the assistance that they need as well as contributing to the work burden of colleagues. When this is combined with implicit or explicit pressure from leadership to ‘just deal with’ secondary trauma and push self-care to the margins, the forced strength that results is fragile and tenuous.

#### 3.2.2. Masking Feelings

Part of Forced Strength is the idea that emotions arising from the work of APW, seizures, and surrenders needed to be hidden, compartmentalized, or managed in a way that did not affect others. When it came to discussing the emotional toll that APW work takes, P4 related that they handled things differently than their colleagues, citing their upbringing and somewhat removed relationship with companion animals. They articulated that “the particulars of situations” did not really “get to” them because they were able to reframe rather than become “gooey mushy” and emotional about a given situation. Instead, they referenced the volume of work as impacting their own work–life balance and well-being. As with P4, P5 stated that she was not affected by compassion fatigue or burnout, despite the “horrible, horrible things” she had seen in the course of her animal welfare work. Instead, she embraced a compartmentalization of emotions as a coping method: “you have to shut yourself off at times emotionally and carry on”. P5 further stated that the inability to keep the emotions contained in any given situation was “a serious problem”, implying that it was not professional and would be a detriment in handling the situation. For P5, masking her emotions was a point of professional pride. P7 followed a similar strategy as P5, that when dealing with a guardian they “steel themselves”, but may be a “blubbering mess” afterwards. The strength for P7 was forced by the need to handle the immediate situation by masking their emotions, understanding that crying in the moment does not help anyone, comparing it to the lack of professionalism of a doctor crying while conveying a cancer diagnosis.

As discussed under Feeling Prepared, multiple participants spoke about their experiences and fear of conflict, and potentially violence, when interacting with animal guardians in emotionally charged situations. P10 spoke about the need to mask feelings in the field, referencing the persistent fear they felt, but had no organization support or resources to help deal with this. There is a forced strength in such situations that APW workers must manage, but this comes at the cost of compassion fatigue and burnout. P10 described herself as a “tough cookie”, never thinking that compassion fatigue would happen to her. She described herself as “a casualty” and that it was “incredibly embarrassing and humiliating to crash and burn”. P10 references the guilt and shame associated with compassion fatigue, and the perception that both compassion fatigue and burnout are associated with not being strong enough. This aspect of forced strength was alluded to by several participants, along with the unwillingness to acknowledge that they were experiencing symptoms of burnout. The idea of ‘just needing to push through’ the stress and negative emotion was common among participants. However, few participants mentioned that open dialogue about their emotions, or raising that they were struggling with secondary trauma, was perceived as possible in their organizations. The fact that participants felt unable to raise issues of compassion fatigue or burnout speaks directly to the stigma that is still attached to mental health, and the forced strength that the stigma engenders. It is this aspect of forced strength that is problematic and that TIP can help to address by creating a safe space for APW staff to discuss their emotions, and establishing open dialogue around secondary trauma and mental health needs. Organizational leadership is pivotal in implementing supportive practices such as debriefing sessions or open dialogue, understanding that these practices reduce stigma around mental health challenges, and can eliminate the forced strength often used in masking negative emotions or secondary trauma.

Masking feelings also arose in the context of the public perception of others. P1 shared that the difficult work is taken home, and that this takes a huge toll on staff: “People just have this expectation that it’s all puppies and kittens and saving lives and I’m like, yeah, on the outside”. P6 shared that they would receive Facebook comments saying “I would love your job being with puppies and kittens all the time”. There is an emotional strength forced by the misperception of others, and the frustration of knowing that most people do not want (or need) to know the difficult situations that APW workers find themselves dealing with. There is also the public perception that APW staff can handle the hard and heartbreaking cases because that is simply what they do. Thus, APW workers feel they have to mask the symptoms of compassion fatigue and burnout. This closes off opportunities to openly talk about mental health challenges that may be occurring with social networks outside APW work, contributing to a sense of isolation. While TIP is not aimed at broad scale social change, in the context of APW work, a trauma-informed approach taken by an organization can normalize and validate these emotional reactions in staff, and facilitate healthier ways to manage secondary trauma by creating a safe environment within the organization and reducing the sense of isolation.

Some participants shared that their organizations had a different vision of what strength looked like in the context of APW work, and one much more in line with TIP. P3 related how mental health and debriefing after a difficult surrender was not taboo, but was in fact encouraged, asserting that “giving people an opportunity to speak freely about some of the situations that have been bothering them in terms of surrendered animals” was an important part of the long-term strength of the organization. It is important to note here that an understanding on behalf of leadership that modelling these healthy behaviours, such as debriefing, is essential in normalizing them. In this sense, the organizational coping strategy of debriefing limited feelings of isolation reduced the impetus to portray a forced strength, and shifted the focus towards building resilience among staff by fostering a solution-oriented approach combined with supportive practices. Resilience represents a healthier aspect of emotional and mental strength rather than mere coping strategies.

When it comes to compassion fatigue and burnout, and their connection to positive mental health and strength, participants were quite forthright about their experiences, including the shifting nature of what strength means. P6 shared that they jumped into their new position after being burned out at their previous job: “I wouldn’t say I’m back to my old strength, but also maybe I will never be able to get that back, who knows, right? So yeah, definitely I’m more fragile some days, but most days I’m absolutely fine. It’s more just we need those moments where we see that our work is actually fruitful and actually changes behaviour”. P6 acknowledged the variability in strength from day to day, but also pointed to a positive way to maintain strength, in looking to the successes in the work, debriefing with trusted colleagues, and engaging in self-care on her days off. 

TIP embraces the honesty that P6′s words evidence. Bad days and tough situations will occur; these days and situations are difficult to deal with, and the range of resulting emotions are validated via the organizational recognition of the trauma inherent in APW work. Strength does not mean a lack of emotion or masking of feelings, but rather the acknowledgment of emotion and its effect on a person. Importantly, TIP encourages identification of the positive as well, and advocates for putting in place ways to validate experiences while offering opportunities to foster resilience and healthy balance. Looking to successes, “the moments where we see the work is actually fruitful”, and feeling as though they are helping humans and animals is a key piece of TIP in APW work. Part of implementing TIP with people accessing services involves meeting the person where they currently are in their life, with no judgement. Meeting staff where they are at, with no judgement around the emotional responses and with an understanding of the limits of individuals in dealing with trauma, is a fundamental aspect of implementing TIP in APW work. Debriefing practices, comprehensive mental health services, modelling by leadership, collective discussion about difficult cases, and wellness days are only a few ideas rooted in TIP, and are all ideas that participants raised in contributing to a healthier work environment for animals, guardians, and staff alike.

## 4. Discussion

The benefits of trauma-informed practices (TIP) have been documented in a variety of fields for service users, and increasingly for personnel in the social services sector [19,20,24,34,44]. The current study explored the challenges experienced by workers in the field of animal protection and welfare services, specifically related to the circumstances of seizures and surrenders. Importantly, the challenges, and the necessary changes to address the challenges raised by participants, are mirrored in other service sectors.

Participants in the current study felt unprepared for the realities of their day-to-day work, including the level of human and animal trauma witnessed, the emotional impact of witnessing such trauma, the overwhelming workload, and the structural elements such as lack of individual or community resources. These are also challenges seen in the support services and emergency services sectors [6,13,19,20,45].

Police-focused research into compassion fatigue, burnout, and work related stresses tends to focus on sworn officers and the trauma that they encounter through the course of their regular duties [8,9,10]; however, similar impacts are felt by civilian employees as well [11,12]. Burnett, Sheard, and St Clair-Thompson surveyed 605 police officers, staff, and volunteers from a police force in the UK, observing that there were high levels of stress, burnout, and compassion fatigue across the sample, though they did not analyze differences based on job roles [12]. McCarty and Skogan examined the difference between civilian and sworn officers in a sample of US police agencies, finding that there was a not a significant difference in burnout between the two police roles [11]. Arluke also included both front line APW workers and dispatchers in his ethnography of animal protection services, finding that both groups experienced cynicism and frustration [15]. Relevant to the current study, which includes animal protection and welfare workers, these three studies did not limit their sample to law enforcers, but included a diversity of job roles within the policing and APW sectors. This is reflective of the role makeup of the current sample of participants, and the APW sector at large, in which multiple roles encounter stressful events, including surrenders and situations that may lead to seizures. TIP is important to mitigate trauma for all workers in APW regardless of role.

Ezell pointed to the secondary trauma experienced by family service workers and the high caseloads as reasons why TIP was critical for both staff and clients [20]. Similarly to the subjects of the current study, family service workers also deal with effects of compassion fatigue and stress due to feeling overworked [20], and the symptoms (taking work home, crying, and emotional outbursts) were similar to the APW participants. High caseloads and their negative effects were also documented in interviews with APW workers in Ontario [23]. One of the recommendations from the resulting report was an increase in labour resources together with a decreased workload as a means to support both AP workers and animals [23]. Recommending a relative reduction in workload is not unique to APW, but is a consistent policy suggestion across sectors with high rates of burnout and compassion fatigue [11,13,20,41]. An adoption of TIP includes a realistic assessment of workload, understanding the trauma that can compound from overwork and lack of time or energy for self-care practices. Given that the AP sector is consistently under-resourced, increasing staff levels represents an organizational-level factor that is a foundational part of adopting TIP. This embodies the key TIP principle of safety grounded in the emotional and mental safety of staff [37].

Family service workers also recognize the lack of resources connected to both individual and community socioeconomic status, and convey frustration over the reduced ability to support clients in what they need [20]. However, there is a key difference in resourcing of APW organizations in that nearly all are non-profit organizations operating with a reliance on charitable donations [1], as opposed to government-funded family services. While a lack of resources may be a barrier to the full adoption of a trauma-informed approach [20], within the APW sector, this can be viewed as a space where embracing TIP can help to mitigate the impacts of an under-resourced community and an overworked staff. Under a One Welfare and trauma-informed approach, collaboration between human and animal service organizations is a way to better support human guardians and their companion animals even with limited resources, creating a network of supports rather than relying on a single support. This addresses another of the key principles of TIP, that of collaboration and mutuality [37].

The lack of resources and feeling overworked contributed to the forced emotional strength of the participants in the current study. This idea of forced strength has appeared in other studies of the APW sector in relation to high caseloads and lack of labour resources [46]. Arluke introduces the concept of humane realism [15], wherein APW workers embrace a realistic understanding of what they can and cannot accomplish within the bounds of their roles. Humane realism is one way in which the forced strength can be managed, through the acknowledgement of the shared end goal of assisting animals in need and working within constraints, such as limited resources and lack of understanding of the difficulty of the job, to accomplish this goal [15]. However, Arluke is also cognizant of the ways in which humane realism is counterproductive, specifically in how “efforts to manage the problems by becoming humanely realistic, [APW workers] end up reproducing and perhaps furthering them” [15] (p. 162). This is akin to how participants in this study navigated feelings of forced strength related to overwork; for example, in rationalizing the inability to take time off because the animals may suffer. In essence, this reproduced the problems they identified by forcing a strength in dealing with the challenges of the job. TIP would be beneficial in identifying and addressing the foundation of these problems, such as high caseloads and limited resources that lead to forced strength, and compromised mental health. TIP would also support building resiliency among APW workers in establishing clear policies and practices that support mental health, which would also be connected to the positive aspects of a humane realism.

One reality of the APW sector is the gendered nature of the work, as care work is predominately performed by women [21,46,47,48], and this is reflected in the sample in the current study. Coulter and Fitzgerald observe how the gendered composition of animal cruelty investigators complicated their jobs and increased work-related stresses. Similar to the participants in our study, the participants in Coulter and Fitzgerald’s research spoke of being unable to show emotion at difficult calls. Such forced strength served a protective function, as showing emotion undermined their authority with involved humans, and forced strength was a way to cope during the difficult and potentially dangerous situation [46]. However, this added the stress of managing not only the physical and emotional safety concerns at a given call, but also the interpersonal dynamic in which insults or threats were directly related to gender-established conditions for compassion fatigue and burnout [46]. This is similar to the current study where participants, mostly female, were subjected to threats and insults. While our sample in the current study is not large enough to perform a gendered analysis, the experiences of participants are reflected in existing research. Other studies have also noted the gendered nature of APW work [21,47,49]. In her study of animal shelter workers, Taylor observed that anger and hostility towards individuals who abused, neglected, or surrendered their animals were generally not well masked in interactions with the public, and empathy and compassion were reserved for the animals [47]. Taylor noted that these emotional displays contradicted the ‘softer emotions’ expected from women, but served a purpose in managing the challenges of APW work. An implementation of TIP should hold space for APW workers to express the range of emotions, acknowledge the unique gendered experiences of staff, and establish processes to support emotional and physical safety.

Training and education are necessary components of a trauma-informed approach [19,35,36]. The need for increased training was a recommendation from all AP participants, both in the context of increased skills to handle conflict as well as tools to support positive mental health. Training and education in preventing compassion fatigue and burnout is echoed both in other works looking at the APW sector [14,27] and human services in general [19,24,34]. Civilian police personnel report inadequate training when compared with their colleagues who are sworn officers, and such inadequate training leads to burnout [11]. Butler and colleagues were surprised that specific training on how to deal with the trauma was needed for students in a Masters of Social Work program, having expected students to be better able to cope given they were drawn to the field [19]. The assumption of being able to cope with the trauma of APW work was also evidenced in the voices of animal protection officers and the perception of others that “only a hardened person, or one that never cared about animals, could do their work” [15] (p. 16). In Schabram and Maitlis’ study, animal shelter workers framed their work as a ‘calling’, with many positioning themselves as “uniquely able to bear the burden of shelter work” and describing themselves as becoming numbed or desensitized to the work as a method of coping [21] (p. 597). This is similar to the forced strength in the current study, with the expectation that because participants chose the APW field, they were able to handle the various challenges of their work. Rault et al. highlight the need for comprehensive and ongoing training for APW, which “include hard skills such as CPR and first aid, while acknowledging the officers’ mental health by offering training and support relating to compassion, fatigue, officer safety, and stress management” [16] (p. 28). While not specifically named as TIP, the inclusion of mental health-focused training and supports related to job stress are components of a trauma-informed approach. The success of implementing TIP in an organization supporting people with mental health and substance use issues was largely attributed to the comprehensive training program, which targeted not only the principles of TIP, but how these principles related to the lived realities of the job [36]. 

Training and education addresses several of the key TIP principles. Firstly, training in communication and recognizing trauma is connected to the key TIP principle of collaboration and mutuality. This can include collaborating with the human guardian on solutions or needed resources, as well as meeting the guardian where they are at without judgement. Collaboration and communication have been shown to be a fundamental part of implementing TIP in domestic violence service settings for both client and worker satisfaction [34,44]. Training and education around communication also addresses the key principle of safety for the guardians, in establishing skills to “promote emotional safety” [34] (p. 593), through non-judgemental communication and an understanding of trauma. This also serves the TIP principle of trustworthiness and transparency. Building trust through open and honest conversation with guardians about the situation, identifying the strengths and needs of the guardian and animal, and collaborating with the guardian to address the situation all lead to more trust. Transparency is an essential aspect of building and maintaining trust with animal guardians, and can be incorporated as a practice by, for example, being open and honest about processes, procedures, and options in a situation. This built trust can serve to reduce conflict. Such skills also increase APW staff safety as stronger communication skills (along with trust building and transparency) reduces the chance of conflict and stressful interactions with guardians.

A component of trauma-informed training and education is to recognize and destigmatize the symptoms of compassion fatigue and burnout in both one’s self and others, and this includes leadership. Aligned with this recommendation is the establishment of organization-level resources and supports for positive mental health. This meets the two key TIP principles of peer support and safety. Some of the ideas raised by participants in the current study are held as best practice in trauma-informed approaches. For example, debriefing with peers and establishing regular check-ins are two practices that are advocated in implementing TIP for those in social service work [35]. Police also report the support of peers as being a welcome resource to deal with difficult situations [10]. For the APW sector, peers could be coworkers or even retired colleagues with a wealth of experience to share. Critically, “organizational trauma-informed structures could include strategies like mental health days, places to exercise or meditate in the work environment, lunch periods that are honored and encouraged, workshops on self-care strategies, or expectations for the workday to conclude once the person leaves the work environment” [35] (p. 6). It is crucial to the success of TIP that the burden for mitigating burnout and compassion fatigue not be placed solely on the workers. For instance, Tuttle et al. suggest organizational change through incorporating clinical mental health workers into a police force (in trainings, routine check-ins, and staff meetings) as a way to destigmatize seeking mental health support [9]. APW organizations could establish consistent resources such as social workers or counsellors with specialized training in compassion fatigue and burnout alongside a grounded understanding of APW work. The key is a commitment on the part of the organization to implement TIP in a fulsome manner, which includes policies and resources. Successful implementations of TIP have substantial organizational change as a foundation [36].

In the context of veterinary practice, the importance of social support in reducing occupational stresses has been noted [50], and a supportive team environment has been observed as crucial in mitigating stress and burnout [51]. A supportive environment can mean different things to different people, and look differently across organizations. For social workers in Kapoulitsas and Corcoran’s research, having an ‘open door’ to debrief with supervisors was an important aspect of a supportive environment [13]. In the context of the current study, support was defined by the participants as the ability to debrief, support for mental health, open communication about secondary trauma, and generally feeling able to share their emotions. The recommendations from the existing literature together with those from participants in the current study speak directly to the key principles of peer support and safety, in creating an organizational environment where coworkers support each other, leadership models positive mental health practices, and everyone feels safe and empowered in embracing a healthy version of strength.

It is vital to understand that TIP is not a ‘one and done’ approach, nor is it a single coping strategy. Rather, it is a multi-pronged approach with resilience and well-being at the core. In order to create a more trauma-informed organization, multiple polices need to be implemented and work in conjunction. This includes the necessity of a fair workload to reduce and prevent burnout [23,51], alongside mental health supports, practices that recognize and mitigate secondary trauma [7,19], and positive modelling by leadership [9,36]. Similar to the process of building and sustaining resilience, embracing TIP in animal protection and welfare work requires sustained and reflective practice with attention to the myriad spaces that trauma can originate from, and reflection on what strategies are, and are not, effective for the staff, guardians, animals, and organization as a whole.

As with all research, there are limitations with the current study. The sample of APW staff was small at only 11 participants. Given the in-depth nature of the interviews, smaller samples are appropriate for this methodological approach. With a small sample, there is the possibility that different perspectives of others in the APW sector have been missed. However, many of the challenges raised by the participants, such as overwork, lack of resources, inability to take time off, masking feelings of burnout, and compassion fatigue have been documented by other researchers [23]. This illustrates that though small in number, the participants voiced common concerns in the APW sector.

The lack of diversity in the sample is another limitation of the project, as the participant sample self-identified as primarily White and female. Given that the majority of workers in APW identify as female [23], our sample is representative of the gender distribution in the field. We believe this limitation is related to two factors: First, the sampling technique that was utilized, in which social media posts and targeted emails were used to seek APW and trauma-informed workers, and potential participants were invited to contact us if they were interested in the study. These techniques pose the limitation of barriers to access, including Internet access, media literacy, and social capital. Second, evidence suggests that in the APW field, workers are predominantly White [52]. A diverse sector is important to better serve a diverse society, and we can accomplish more through including different perspectives [52]. This points to the broader importance of addressing diversity, equity, and inclusion in the APW sector. Future research should keep in mind the need for diversity, and employ sampling strategies that better capture the existing diversity within the field.

Activities leading from this study include a document analysis and development of TIP-related training modules. We had initially planned a content analysis of surrender and seizure policies, documents (e.g., surrender forms and information provided to guardians), and standard operating procedures; however, given the small sample, we were unable to obtain sufficient material for analysis. A documentary analysis is planned as the follow up to the current study. We have also developed three training modules for TIP for the APW sector: one on self-care and recognizing and preventing compassion fatigue and burnout, one on implementing a trauma-informed and culturally safe approach in an organization, and one specifically for leadership on implementing TIP grounded in organizational change. A follow up survey and evaluation processes to assess the success of the training are being planned. 

## 5. Conclusions

This study presents very clear benefits, including highlighting how TIP is critical for the APW sector, and gives voice to the challenges within the APW sector. Ideally, TIP leads to positive transformation and provides a strong individual, interpersonal, and organizational foundation to prevent compassion fatigue and burnout. This not only helps the APW worker in managing job-related stress and trauma, but also increased compassion for animals and their guardians, leading to a more compassionate experience for everyone. APW workers feel better about their job, which means a stronger, more resilient, more consistent organizational face in the community. TIP is a critical component in facilitating relationship building with the communities that the APW organization serves.

P2 provided an excellent summary of why TIP is needed in APW:

“We’re not gonna [sic] lose our empathy for animals. Animals are why we do what we do, animals are not held to blame, you know. It is people that we lose empathy for. And I think that is a problem, can become a problem especially if you’re dealing with people who already have trauma”.

TIP includes AWP workers in those who experience trauma as part of their job, and seeks to foster empathy for others as well as one’s self. In short, implementing TIP in APW is crucial for the health of everyone, human and animal alike.

## Data Availability

The data are not publicly available due to confidentiality and privacy of the participants connected to the nature of the qualitative interview approach combined with the small sample.

## Appendix A

This article reports on a subsection of a larger project; the interview guide presented here is also presented in [18].

1. What is the gender with which you identify?
2. What is the primary race/ethnicity with which you identify?
3. How old are you? You can even me an approximate decade if you’d prefer.
4. How would you identify the area that your agency is located in? (rural/remote or urban)
  - Follow-up: do you service clients that are in rural/remote areas?
5. What is your position within your agency?
6. Could you talk to me a bit about your process of becoming a [job description]
7. Could you talk to me a bit about your experience as a [job description]?
8. Could you talk a bit about what it’s like to work at [agency]?
9. What services are available to the clients at your agency?
10. What is your role in regard to the process of the surrender or seizure of animals?
  - Probe: What is the process of interacting with clients in these instances? (e.g., non-judgmental approach, learning opportunity, etc.). Is there training of staff or volunteers around this?
  - Probe: What are your thoughts on which approaches or policies seem to be working well, specifically in regard to the process of the surrender or seizure of animals?
11. What do you think the biggest barriers are for yourself and your agency when assisting clients with their pets who have health and welfare-related needs?
  - Probe: What do you think could be improved in regard to assisting clients with their pets who have health and welfare-related needs?
12. How are you being supported in your role? What do you wish you were provided with that might help better support you?
13. Have you heard of the term ‘compassion fatigue’?
  - [If yes]: Do you feel that this is something you have experienced with your work?
  - [If no]: Explain concept and probe to see if participant has experienced this.
14. How about the term ‘burnout’, is that something you have heard of before?
  - [If yes]: Do you feel that this is something you have experienced with your work?
  - [If no]: Explain concept and probe to see if participant has experienced this.
15. Do you have any employment assistance programs available to you?
16. How adequate are the supports/services provided to yourself and other workers in processing and coping with the sometimes-difficult things you might see or experience in your role?
17. Is there anything else that you feel is important for me to know about yourself, or the work that you do at your agency?
18. Before we finish up, do you have any questions for me related to the study or interview?
